# Almond (*Prunus dulcis*) Bagasse as a Source of Bioactive Compounds with Antioxidant Properties: An In Vitro Assessment

**DOI:** 10.3390/antiox12061229

**Published:** 2023-06-07

**Authors:** Stevens Duarte, Almudena Puchades, Nuria Jiménez-Hernández, Ester Betoret, María José Gosalbes, Noelia Betoret

**Affiliations:** 1 Instituto Universitario de Ingeniería de Alimentos para el Desarrollo, Universitat Politècnica de València, 46022 Valencia, Spain; steduase@doctor.upv.es (S.D.); almudena9903@hotmail.com (A.P.); mesbeval@upvnet.upv.es (E.B.); 2 Fundación para el Fomento de la Investigación Sanitaria y Biomédica de la Comunidad Valenciana-Salud Pública, 46020 Valencia, Spain; jimenez_nurher@gva.es; 3 CIBER de Epidemiología y Salud Pública, Instituto de Salud Carlos III, 28029 Madrid, Spain

**Keywords:** bioactive compounds, almond by-product, antiradical capacity, in vitro digestion, colonic fermentation, gut microbiota, metagenomics

## Abstract

The presence of components of nutritional interest makes fresh almond bagasse an interesting by-product for obtaining functional ingredients. Stabilization through a dehydration process is an interesting option for its integral use, ensuring its conservation and management. Subsequently, it can be turned into powder, facilitating its use as an ingredient. The aim of this paper was to determine the effects of hot air drying at 60 and 70 °C and lyophilization on the release of phenolic components and antiradical capacity in in vitro gastrointestinal digestion and colonic fermentation, as well as on growing microbiota composition by applying high throughput sequencing. The novelty of this study lies in this holistic approach; considering both technological and physiological aspects related to gastrointestinal digestion and colonic fermentation will provide the best conditions for functional foods. The results obtained showed that lyophilization provides a powder with a total phenol content and antiradical capacity higher than hot air drying. Furthermore, in dehydrated samples, both in vitro digestion and colonic fermentation revealed a phenol content and anti-radical capacity superior to those existing in undigested products. In addition, after colonic fermentation, beneficial bacteria species have been identified. Obtaining powders from almond bagasse is presented as an interesting opportunity for the valorization of this by-product.

## 1. Introduction

Consumer trends and the agri-food industry have evolved due to more sustainable and nutritional diets [1]. Plant-based products are being extensively assessed as sustainable alternatives to animal protein and as important sources of bioactive compounds [2]. The consumption of cereal, legume, or nut beverages such as oat, soy, or almond vegetable drinks has been increased in recent years [3]. Particularly, in Mediterranean countries, the almond (*Prunus dulcis*) vegetal drink has deserved particular attention since it serves as an excellent source of dietary fibers, monounsaturated fatty acids, vitamin E, protein, essential minerals, riboflavin, and antioxidants [4]. Extracts of whole almond seed have demonstrated to possess potent free radical scavenging capacities [5]. These activities have been related to the presence of flavonoids and other phenolic compounds [6].

During the process of obtaining the almond beverage, many components of the kernel are solubilized and transferred to water in a solid–liquid extraction process. However, a considerable amount is retained in the generated solid residue known as bagasse or cake. Although there are many studies determining the contents of macronutrients and bioactive compounds in the kernel, hull, and shell, no studies have been found providing a detailed polyphenolic composition of almond bagasse [6].

On the other hand, the large amount of bagasse generated needs to be managed in order to avoid an important ecological footprint. Finding high-value-added alternatives beyond animal feed is a challenge. Due to its high water activity, recovery must first include the stabilization of the product. Otherwise, microorganism spoilage and rancidity can become a problem, leaving it useless. The combination of grinding and dehydration has been shown to be a suitable processing method for the production of stable powdered ingredients with interesting industrial applications [7]. This form of processing has been applied to a wide range of fruits and vegetables and there are now many powdered products on the market. In recent years, research in this area has also been extended to several by-products, in particular the effect of processing on the content and bioaccessibility of bioactive compounds has been studied. It is well known that processing conditions can affect the structure, functionality, and accessibility of bioactive compounds, determining their beneficial effect. Furthermore, its particle size and the presence of other macromolecules such as carbohydrates or proteins could influence chemical or physical interactions, facilitating or inhibiting the release and absorption of bioactive compounds in the gastrointestinal tract [7].

Furthermore, much research is being conducted to elucidate the influence of diet on the colonic fermentative microbiota since it plays a fundamental role in the degradation of complex macromolecules and affects the metabolism of phenolic compounds and secondary metabolite generation with a potential beneficial effect on human health [8]. However, in order to ensure the best health effects, the effect of processing, the evolution along the gastrointestinal tract, and the influence on the colonic fermentative microbiota must be analyzed as a whole. A holistic approach, considering both technological and physiological aspects related to gastrointestinal digestion and colonic fermentation, will provide the best conditions for functional foods. The novelty of this work lies in this holistic approach, including the effects of processing, gastrointestinal digestion, and colonic fermentation.

The aim of this paper was to examine the effect of dehydration conditions and in vitro gastrointestinal digestion on antiradical capacity, total phenols, and specific polyphenol content in almond bagasse. Moreover, the effects on the fermentative microbial community and the polyphenol content after colonic fermentation have been determined.

## 2. Materials and Methods

### 2.1. Production of Almond Bagasse and Powder

Natural almonds were purchased from a local supermarket in Valencia city (Spain) and ground with drinking water at a ratio of 1/9 (*w*/*w*). Grinding was performed using a domestic kitchen appliance (Thermomix^®^, Vorwerk, Spain) at 10,000 rpm for 20 s. The grind was then filtered, and therefore the almond bagasse was recovered for further characterization and processing.

To obtain the almond bagasse powder, the almond bagasse was homogeneously distributed in plastic grids with a nominal spacing of 2 mm, dehydrated to a water activity lower than 0.3, and finally ground. A convective dryer (Pol-eko Aparatura, Katowice, Poland) with cross-flow air at 60 or 70 °C, for 10 h and 7 h, respectively, was used to produce hot air dried bagasse (HAD), and a freeze dryer (Telstar, Lioalta-g) was used to produce lyophilized one (LYO) from fresh almond bagasse previously frozen at −40 °C for 24 h. Once almond bagasse was dehydrated, the particle size was reduced employing a kitchen appliance (Thermomix^®^, Vorwerk, Spain). Grinding was carried out at 4000 rpm for 20 s at 5 s intervals followed by 10,000 rpm for 20 s at 5 s intervals to obtain almond bagasse powder with a coarse granulometry. Finally, powders were stored in opaque glass jars at room temperature for two weeks maximum.

### 2.2. Simulation of Gastrointestinal Digestion In Vitro

**Figure 1:**
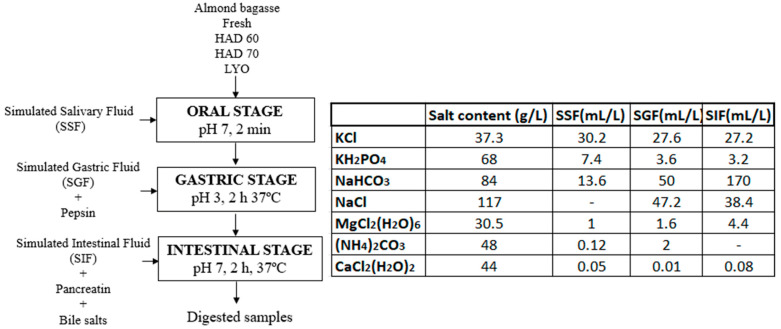
Flowchart, process conditions, and composition of the simulated phases for the different stages of the in vitro gastrointestinal digestion process. HAD60: hot air dried powder at 60 °C, HAD70: hot air dried powder at 70 °C, and LYO: lyophilized powder.

The methodology proposed by Minekus et al. [9] was followed for the simulation of the oral, gastric, and intestinal digestion stages. According to the protocol, the phases mixed in the successive stages must be kept in a 1:1 ratio (*v*/*v*). In this case, 5 g of sample, 5 mL of simulated salivary fluid (SSF), 10 mL of simulated gastric fluid (GSF), and 20 mL of simulated intestinal fluid (SIF) were added. The conditions and procedure are shown in Figure 1. Three repetitions for each type of sample (fresh almond bagasse (FRESH), hot air dried powder at 60 °C (HAD60), hot air dried powder at 70 °C (HAD70), and lyophilized powder (LYO) were performed.

### 2.3. In Vitro Colonic Fermentation

Fecal samples were collected from healthy donors who had not taken antibiotics, prebiotics, or probiotics for three months prior to the assay. The study was conducted according to the guidelines of the Declaration of Helsinki and the subjects gave their informed consent before they participated in the study. The fecal samples from the donors were pooled together to reduce intra-individual daily variability, and the pool was used to prepare the inoculum (10% *w*/*v*). After the in vitro gastrointestinal digestion of the different samples, fresh bagasse (bagasse), hot air dried powder at 60 °C (HAD60), hot air dried powder at 70 °C (HAD70), lyophilized powder (LYO), the solid residue (fraction not available for absorption) that was left after removing the supernatant, plus 10% of such digestion supernatant were used as the substrate (1% *w*/*v*) for fermentation, as described in [10]. The colonic fermentation was carried out in triplicate. Moreover, a control fermentation without a substrate was performed.

Aliquots were removed from the fermenters at baseline (t = 0 h) and after 24 h for further analysis. The incubation and processing procedures were carried out under anaerobic conditions in an anaerobic jar or an anaerobic chamber.

### 2.4. DNA Extraction, Sequencing, and Microbial Analysis

Total DNA was extracted from fermentation aliquots in the robotic workstation MagNA Pure LC Instrument (Roche) using the MagNA Pure LC DNA isolation kit III (Bacteria, Fungi) (Roche), following the manufacturer’s instructions. The V3-V4 hypervariable region of the 16S rRNA gene was amplified using microbial genomic DNA as template, following the Illumina protocol for 16S Metagenomic Sequencing Library Preparation. Sequencing was performed with the Kit v3 (2 × 230 cycles) in a MiSeq platform (Illumina) at FISABIO-Salud Pública. All the sequences have been deposited in the EBI database under the study number PRJEB61665 with the following sample accession numbers: ERSI4956700, ERSI4956701, ERSI4956702, ERSI4956703, ERSI4956704, ERSI4956705, ERSI4956706, ERSI4956707, ERSI4956708, ERSI4956709, ERSI4956710, ERSI4956711, ERSI4956712.

16S rRNA gene reads with a low-quality score and short read length as well as potential chimeras were removed using the DADA2 pipeline in the R package [11]. Additionally, reads were aligned against the human genome (GRCh38.p13) using Bowtie2 (v2.3.5.1) [12] and matches were discarded. The DADA2 pipeline was also used to create the amplicon sequence variants (ASV). The taxonomic information of the 16S rDNA sequences was obtained by similarity comparison using the Basic Local Alignment Search Tool (BLAST) algorithm against the SILVA database (v.138) [13,14]. The analysis of bacterial composition was performed at the genus level and the PERMANOVA tests were performed using the adonis function from the vegan library of the R package with 600 permutations and the Benjamini–Hochberg procedure for false discovery rate control [15]. Bar plots and canonical correspondence analysis (CCA) were generated with in-house R scripts.

The analysis of compositions of microbiomes with bias correction (ANCOMBC) package [16] was applied to identify differentially abundant taxa among substrate fermentations, and test significance was determined using the Benjamini–Hochberg procedure for false discovery rate control (*q*-value).

### 2.5. Determination of Antioxidant Compounds and Antiradical Capacity

For the extraction of antioxidants, 10 mL of a methanol–water mixture was used as a solvent in a ratio of 80:20 (*v*/*v*) and mixed with 1 g of sample. After 1 h of magnetic stirring, it was centrifuged (Selecta, “Medrifriger BL-S”) at 10,000 rpm for 5 min at 20 °C. Determinations were performed on the supernatant which will be referred to as the extract below.

#### 2.5.1. Total Phenol Content

The determination of total phenols was carried out following the colorimetric method of Folin–Ciocalteu [17]. In a spectrophotometric cell, 0.125 mL of extract, 0.125 mL of the Folin–Ciocalteu reagent (Sigma-Aldrich), and 0.5 mL of bidistilled water were added in that order and the mixture was allowed to react for 6 min. After this time, 1.25 mL of 7% sodium carbonate (*w*/*v*) and 1 mL of distilled water were added. As a reference, the extract was replaced by bidistilled water, and allowed to react for 90 min. After that, the absorbance was measured at 765 nm in a spectrophotometer (Thermo Scientific Helios Zeta U/Vis, Loughborough, UK). The results obtained were compared with a standard curve of gallic acid (purity ≥ 98%).

#### 2.5.2. Antiradical Capacity by DPPH and ABTS Methods

The antioxidant capacity was determined following the DPPH method described by Stratil et al. [18] and Kuskoski et al. [19], with some modifications. Specifically, 0.1 mL of the extract and 2.9 mL of the methanol–DPPH solution were mixed and the absorbance was measured at 517 nm in a spectrophotometer (Thermo Scientific Helios Zeta U/Vis, Loughborough, UK). Trolox was used as the reference standard antioxidant (C_14_H_18_O_4_, purity ≥ 7%, Sigma-Aldrich) and a Trolox calibration curve was obtained for the range of concentrations between 0 and 500 mg/L. The results are expressed as milligrams of Trolox equivalent per gram of dry matter (mg TE/g dm).

The antioxidant activity was also evaluated following the ABTS radical method (2,20-azobis-3-ethyl benzothiazolin-6-sulfonic acid). The methodology proposed by Re et al. [20] was followed. A solution of the acid (7 mM, purity ≥ 99%) was prepared with potassium persulfate (2.45 mM, purity 99.99%) in distilled water, and incubated in darkness at room temperature for 16 h. After that, a dilution with phosphate buffer was made until an absorbance of 0.70 ± 0.02 at 734 nm was reached. Then, in a spectrophotometry cell, 0.1 mL of extract with 2.9 mL of ABTS solution was reacted. As a reference, the sample was replaced with double distilled water. The absorbance was measured after 0, 3, and 7 min of reaction time at a wavelength of 734 nm in a spectrophotometer (Thermo Scientific Helios Zeta U/Vis, Loughborough, UK).

#### 2.5.3. Phenolic Compounds by HPLC Analysis

Phenolic compounds were extracted following the methodology proposed by Caprioli et al. [21] and Giusti et al. [22]. To perform and acid hydrolysis, 2.5 g of sample was mixed with 7.5 mL of solvent (70:30 ethanol and double distilled water), the pH was adjusted to 4 with 2 N hydrochloric acid, and transferred to an ultrasonic bath (J.P. Setecta, 3000840) for 2 h at room temperature. The samples were centrifuged at 8000× *g* for 15 min, and the extraction was repeated in the solid sample. The supernatants from the two extractions were filtered with a 20 µm PTFE filter and subsequently analyzed via HPLC.

For a basic hydrolysis, 14 mL of a solution of 2 N sodium hydroxide, 0.01% EDTA 10 mM, and 0.1% ascorbic acid was mixed with the pellet obtained after the acid hydrolysis and left overnight to facilitate the release of phenolic ethers or esters. The pH was adjusted to 2 with 6 N hydrochloric acid and centrifuged at 8000× *g* for 15 min. The supernatant was mixed with 15 mL of a 50:50 ethyl acetate/diethyl ether solution and centrifuged at 5400× *g* for 15 min twice. The supernatants were collected and concentrated on a rotary evaporator (Heidolph) at 26 °C. The resultant solution was reconstituted with 10 mL methanol and filtered through a 20 µm PTFE filter. Finally, the phenolic fraction was analyzed via HPLC.

The samples were processed using a 1200 Series Rapid Resolution HPLC coupled to a Series diode array detector (Agilent, Palo Alto, CA, USA), according to the methodology described by Tanleque-Alberto et al. [23]. Phenolic extracts were separated on a Brisa-LC 5 column Tanleque-Alberto et al. [23]. Mobile phase B was acetonitrile and mobile phase A was 1% formic acid. The gradients were 0 min 10% B, 25 min 60% B, 26 min 80% B holding until 30 min, and 35 min 10% B holding until 40 min. The working conditions were 30 °C, 0.5 mL/min injection volume, and 10 µL flow rate. The different phenolic compounds were identified with the chromatographic retention times of reference standards for each compound at the following wavelengths: vanillin, 250 nm; 260 nm for 4-hydroxybenzoic acid, rutin, and quercetin 3-glucoside; 280 nm for epicatechin and chlorogenic acid; 320 nm for *p*-coumaric acid, sinapic acid, ferulic acid, and apeigenin-7-glucoside. Specific compounds were quantified using calibration curves and the results were expressed in mg/100 dry sample.

### 2.6. Statistical Analysis

All determinations were performed in triplicate. The statistical analysis of the data was performed in a Statgraphics Centurion XVII Software Package, using a simple or multifactorial analysis of variance (ANOVA) at a 95% confidence level (*p* ≤ 0.05). Significant differences among groups were determined using the Fisher LSD test.

The PERMANOVA test, based on a dissimilarity test, was applied to evaluate the effect of the external factors on the bacterial composition using the adonis function with 600 permutations from the vegan library of the R package. The Benjamini–Hochberg procedure was applied for false discovery rate control.

## 3. Results and Discussion

### 3.1. Effect of Processing on Antiradical Capacity and Phenol Content

In this work, ten of the most common specific polyphenols found in almond kernel were determined in the almond bagasse. The ten polyphenols determined belong to the next three groups: hydroxybenzoic acid derivatives (vanillic acid, 4-hydroxybenzoic acid), hydroxycinnamic acid derivatives (*p*-coumaric acid, sinapic acid, ferulic acid and chlorogenic acid), flavonoids, and derivatives (rutin, quercetin-3-glucoside, quercitrin, epicatechin and apigenin-7-glucoside). Epicatechin was the polyphenol with the highest content (732.7 ± 1.4 mg/100 g dry matter) in fresh bagasse followed by vanillic acid (292.3 ± 6.9 mg/100 g dry matter), rutin (254.7 ± 1.4 mg/100 g dry matter), and sinapic acid (208.4 ± 0.9 mg/100 g dry matter).

**Figure 2:**
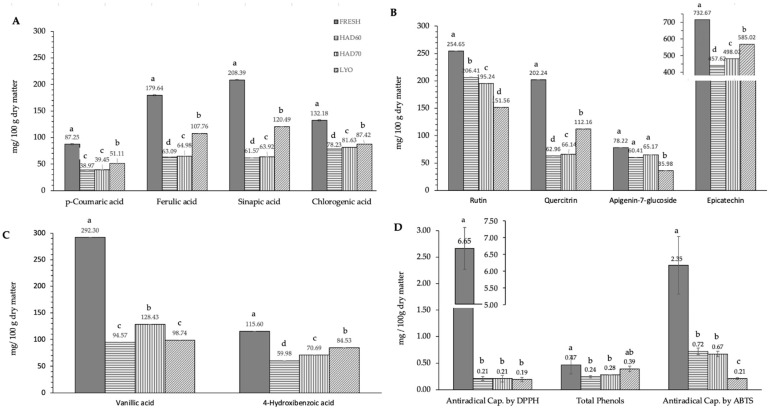
Effect of dehydration treatment on the polyphenols content and antiradical capacity. Phenolic compounds have been grouped according to their chemical structure: (**A**) hydroxycinnamic acid derivatives (*p*-coumaric acid, sinapic acid, ferulic acid and chlorogenic acid), (**B**) flavonoids and derivatives (rutin, quercetin-3-glucoside, quercitrin, epicatechin and apigenin-7-glucoside), (**C**) hydroxybenzoic acid derivatives (vanillic acid, 4-hydroxybenzoic acid). Section (**D**) includes antiradical capacity determined using DPPH and ABTS methods and total phenol content. FRESH: fresh almond bagasse; HAD60: hot air dried powder at 60 °C; HAD70: hot air dried powder at 70 °C; LYO: lyophilized powder. In section 2D, DPPH and ABTS results are expressed as mg Trolox/100 g dry matter; total phenols are expressed as mg galic acid/100 g dry matter. Different superscript letters mean statistically significant differences (*p* ≤ 0.05).

The effect of dehydration treatment on the polyphenol content is shown in Figure 2. Treatment always had a significant effect on the content of polyphenols (*p* ≤ 0.05). The effect was different depending on the specific polyphenol considered. In almost all cases, hot air drying resulted in a bigger degradation of polyphenols than lyophilization, except for rutin (Figure 2B) and apigenin-7-glucoside (Figure 2B) flavonoids in which lyophilized samples (151.6 ± 0.9; 36.0 ± 16.2) resulted in a lower content than hot air-dried ones (200.8 ± 0.6, 62.8 ± 10.4). Regarding hot air drying, the temperature affected polyphenol content significantly (*p* ≤ 0.05). Generally, hot air drying at 60 °C resulted in a lower content of polyphenols than hot air drying at 70 °C. However, in the case of rutin flavonoid (Figure 2B), the degradation was bigger when hot air drying at 70 °C (195.2 ± 0.2) than when hot air drying at 60 °C (206.4 ± 0.9). The overall trends observed in the ten specific polyphenols analyzed were confirmed for the total phenol content (Figure 2C). Processing had a significant effect (*p* ≤ 0.05) on the total phenol content and the effect was bigger in hot air drying at 60 °C (0.243 ± 0.008), followed by hot air drying at 70 °C (0.279 ± 0.014) and lyophilization (0.4 ± 0.2). The total antiradical capacity (Figure 2C) also was affected in a significant way (*p* ≤ 0.05) by processing. In DPPH results, there were no significant differences (*p* ≤ 0.05) among the drying treatments. In ABTS results, there were significant differences (*p* ≤ 0.05) among treatments resulting in lower antiradical capacity in lyophilized samples (0.21 ± 0.06), followed by hot air dried at 70 °C (0.67 ± 0.02) and hot air dried at 60 °C (0.72 ± 0.05).

### 3.2. In Vitro Gastrointestinal Digestion

**Figure 3:**
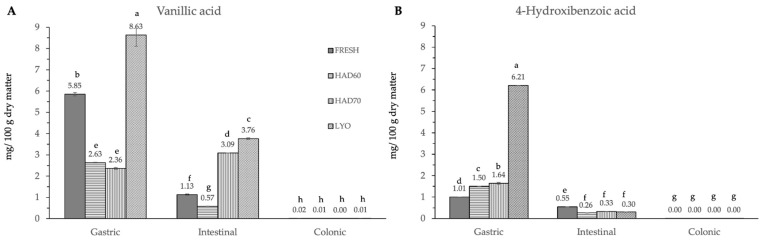
Hydroxybenzoic acid derivatives ((**A**) vanillic acid, (**B**) 4-hydroxybenzoic acid) content after gastric, intestinal, and colonic stages of in vitro gastrointestinal digestion. FRESH: fresh almond bagasse; HAD60: hot air dried powder at 60 °C; HAD70: hot air dried powder at 70 °C; LYO: lyophilized powder. Different superscripts letters mean statistically significant differences (*p* ≤ 0.05).

**Figure 4:**
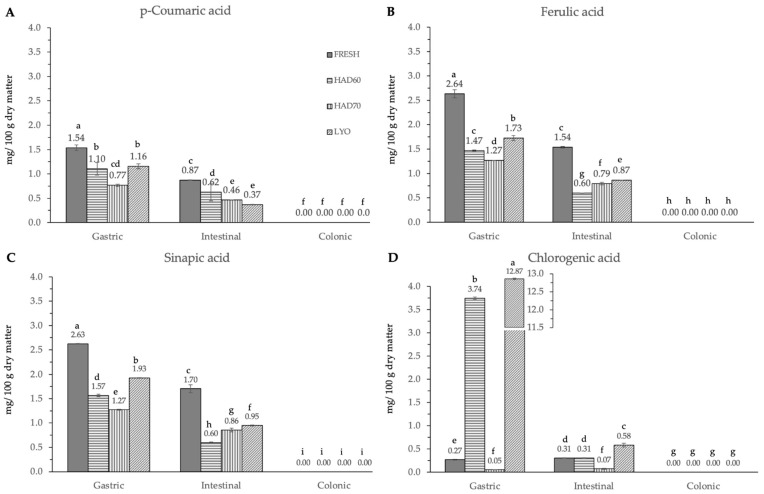
Hydroxycinnamic acid derivatives ((**A**) *p*-coumaric acid, (**B**) ferulic acid, (**C**) sinapic acid and (**D**) chlorogenic acid) content after gastric, intestinal, and colonic stages of in vitro gastrointestinal digestion. FRESH: fresh almond bagasse; HAD60: hot air dried powder at 60 °C; HAD70: hot air dried powder at 70 °C; LYO: lyophilized powder. Different superscripts letters mean statistically significant differences (*p* ≤ 0.05).

**Figure 5:**
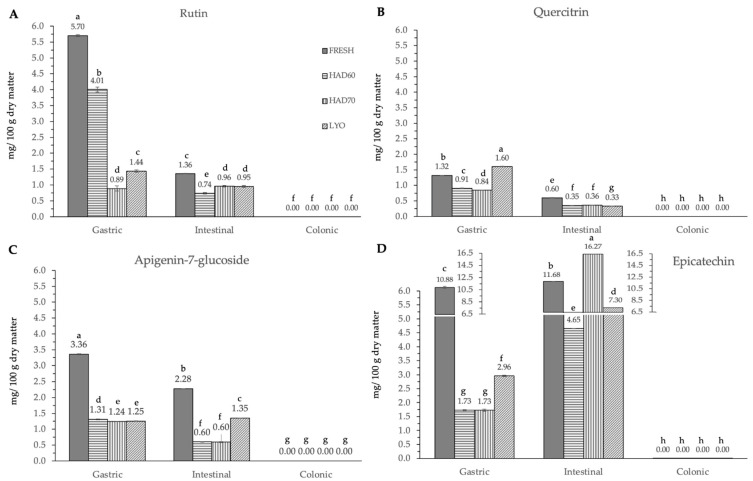
Flavonoids and derivatives ((**A**) rutin, (**B**) quercetin-3-glucoside, quercitrin, (**C**) apigenin-7-glucoside and (**D**) epicatechin) content after gastric, intestinal, and colonic stages of in vitro gastrointestinal digestion. FRESH: fresh almond bagasse; HAD60: hot air dried powder at 60 °C; HAD70: hot air dried powder at 70 °C; LYO: lyophilized powder. Different superscripts letters mean statistically significant differences (*p* ≤ 0.05).

In vitro gastrointestinal digestion caused a significant effect (*p* ≤ 0.05) in all the phenolic compounds determined. This effect was different depending on the considered polyphenol. Although evolution along simulated digestion was variable, the in vitro digestion resulted in a significant reduction in the specific polyphenol compounds. The absence of the considered molecules after the colonic fermentation was remarkable (Figure 3, Figure 4 and Figure 5).

In the gastric stage, the enzyme mixture and acidic conditions significantly reduced the content of most of the components. Specifically, for hydroxycinnamic acid derivatives (Figure 5) and flavonoids (Figure 4), the content determined was higher in fresh samples. However, in the case of chlorogenic acid (Figure 4D) and quercitrin (Figure 5B), the higher content was determined in lyophilized ones (12.87 ± 0.02). Hot air drying had, mostly, a similar effect on the polyphenols determined regardless of the air temperature, with the exception of chlorogenic acid. The intestinal enzyme mixture significantly reduced (*p* ≤ 0.05) the content of polyphenols determined, except for epicatechin (Figure 5D). The content of epicatechin increased in a significant way (*p* ≤ 0.05) after the intestinal stage in the samples after all the treatments and in the fresh ones. The results are similar to those published by Curvas-Limón et al. [24] for the epicatechin from *Aloe Vera*. In our case, the increase was highest in hot air dried samples at 70 °C (16.27 ± 0.04), followed by lyophilized (7.30 ± 0.03), hot air dried at 60 °C (4.65 ± 0.02), and fresh ones (11.7 ± 0.2) (Figure 5D).

**Figure 6:**
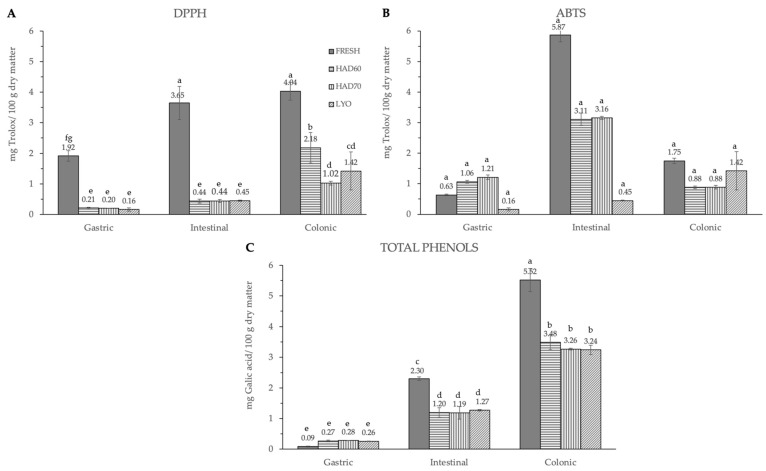
Antiradical capacity by DPPH (**A**), ABTS (**B**) methods and total phenols (**C**) after gastric, intestinal, and colonic stages of in vitro gastrointestinal digestion. FRESH: fresh almond bagasse; HAD60: hot air dried powder at 60 °C; HAD70: hot air dried powder at 70 °C; LYO: lyophilized powder. DPPH and ABTS results were expressed as mg Trolox/100 g dry matter. Total phenols were expressed as mg galic acid/100 g dry matter. Different superscripts letters mean statistically significant differences (*p* ≤ 0.05).

The significant (*p* ≤ 0.05) and progressive increase observed in the total content of phenolic compounds and in both measurements of antiradical capacities after gastric, intestinal, and colonic stages was remarkable (Figure 6). The results were different depending on the reagent used. The total phenolic compounds (Figure 6C) increased in a significant way (*p* ≤ 0.05) at gastric, intestinal, and colonic stages. Although at the gastric stage the total phenolic content of fresh samples was the lowest (0.09 ± 0.01), it significantly increased (*p* ≤ 0.05) at the intestinal (2.30 ± 0.06) and colonic stages (5.5 ± 0.4). There were no significant differences (*p* ≤ 0.05) among the three dehydration treatments, regardless of the digestion stage. In DPPH methodology (Figure 6A), the highest antiradical capacity (*p* ≤ 0.05) was found in fresh samples (6.7 ± 0.6). The antiradical activity increased at gastric, intestinal, and colonic stages. There were no significant differences (*p* ≤ 0.05) found among the values obtained in the gastrointestinal stage with the three dehydration treatments. The same trends observed in the antiradical capacity values measured by the DPPH method and the total phenolic content are probably due to the higher sensitivity of both determinations to low hydrophilic compounds. In ABTS methodology, the highest content (*p* ≤ 0.05) was found at the intestinal stage in fresh samples (5.9 ± 0.2), decreasing again in the colonic stage (1.75 ± 0.08). Among the three dehydration treatments, the values obtained by lyophilized samples were significantly different (*p* ≤ 0.05) and increased at each gastrointestinal stage (0.16 ± 0.05), (0.45 ± 0.02), (1.42 ± 0.62).

### 3.3. Fermentative Microbiota Analysis

Sequencing of the 16S rRNA gene amplicons of the bacterial communities resulting from the in vitro fermentations of fresh almond bagasse, hot air dried powder at 60 °C (HAD60), hot air dried powder at 70 °C (HAD70), lyophilized powder (LYO), and controls were performed. The processing of raw reads (1,494,886) yields 1,173,186 sequences, averaging 86,284 genera per sample. The taxonomic assignment was performed at the genus level.

**Figure 7:**
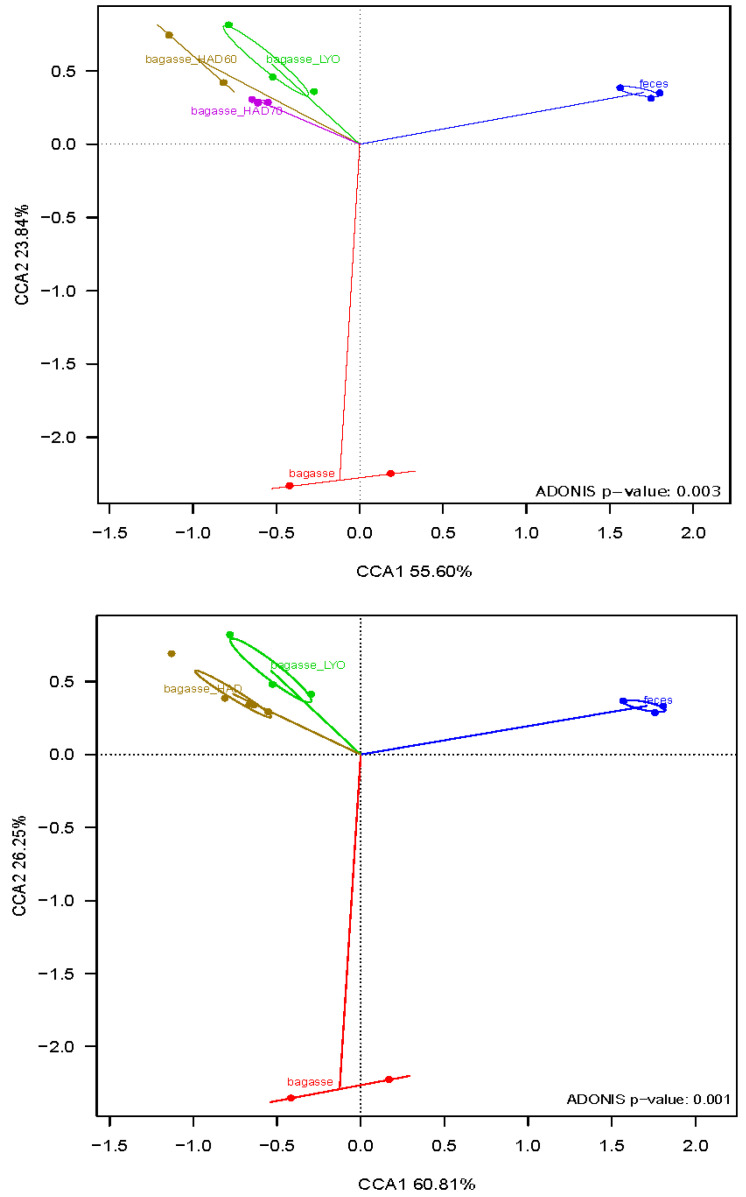
Canonical correspondence analyses (CCAs) at genus level of the bacterial community after fermentations. Bagasse: fresh almond bagasse; bagasse HAD60: hot air dried powder at 60 °C; bagasse HAD70: hot air dried powder at 70 °C; bagasse LYO: lyophilized powder; feces: controls.

Canonical correspondence analysis (CCA) and the adonis test (Figure 7) revealed a significant difference in the microbial community structures. As no significant differences were detected in the fermentative microbiota of air dried powder at 60 °C (HAD60) and air dried powder at 70 °C (HAD70) (*p*-value = 0.4), they were considered as a single air drying treatment for the analysis. The CCA showed the first axis, which explained 55.60% and 60.81% of the variability (Figure 7, and separated substrate-fermentation microbiota and controls (fermentations without substrates). The control overgrowth would be due to residual nutrients in the fecal samples used as inoculum. The second axis, explaining 23.84% and 26.25% of the variability, separated the bacterial community that grew on fresh bagasse from that which fermented the heat-treated (HAD) or lyophilized one (LYO).

**Figure 8:**
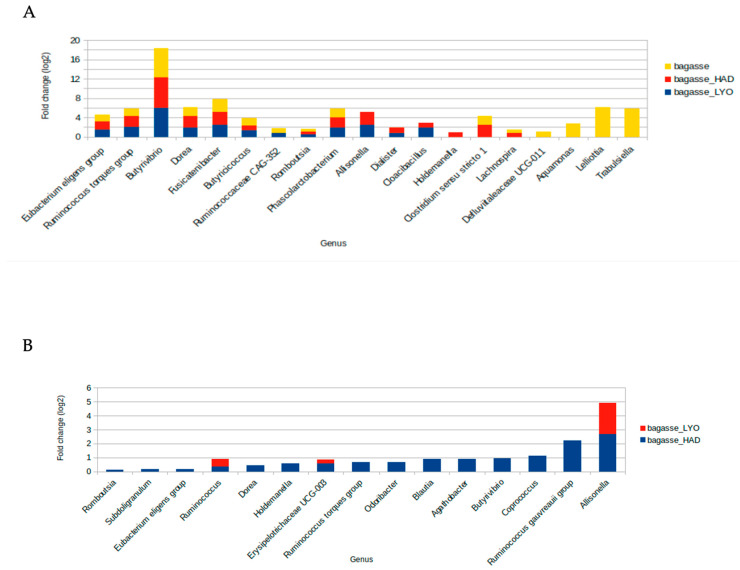
Bar plot of fold-changes of bacterial genera that are significantly increased in (**A**) pairwise comparisons with feces fermentations and in (**B**) pairwise comparisons with the fresh bagasse fermentations. Freeze-dried bagasse (bagasse_LYO), air-dried bagasse (bagasse_HAD), and fresh bagasse (bagasse).

To assess which bacteria were preferentially growing on air-dried, lyophilized, and fresh bagasse, pairwise comparisons, with the feces fermentations as controls, using the ANCOMBC package (Figure 8 and Figure S1) were performed. Thus, it was found that eight genera were differentially abundant after the bagasse fermentations, with *Butyrivibrio* from the Lachnospiraceae family being the genus that presented the greatest increase in abundance in comparison with feces fermentations (Figure S1 and Table S1). The other bacterial groups found at higher abundance in bagasse fermentations were *Eubacterium eligens* group, *Ruminococcus torques* group, *Fusicatenibacter*, *Dorea* from Lachnospiraceae; *Romboutsia* from Peptostreptococcaceae; *Butyricicoccus* from Butyricicoccaceae, and *Phascolarctobacterium* from Acidaminococcaceae (Figure S1 and Table S1). Both dehydration techniques, air-dried and freeze-dried, favor the growth of *Allisonella* and *Dialister* belonging to the Veillonellaceae family as well as the pathobiont *Cloacibacillus* from Synergistaceae. Additionally, *Lachnospira* and *Clostridium sensu stricto* 1 were more abundant after air-dried and fresh bagasse fermentations. Likewise, *Holdemanella* presented higher abundance after air-dried bagasse fermentation. Three Enterobacteriaceae genera and Defluviitaleaceae UCG-011 presented more abundance after fresh bagasse fermentation. In order to investigate the effect on the microbiota composition of the dehydration treatment, ANCOMBC analysis was applied for pairwise comparisons between fresh bagasse, bagasse_HAD, and bagasse_LYO (Figure 8B and Figure S2, Table S2). Both heat-treated and freeze-dried bagasse fermentations resulted in a microbiota with an increased abundance of *Allisonella* (bagasse_HAD, *q* = 0.0002; bagasse_LYO, *q* = 4.1547 × 10^−15^), *Ruminococcus* (bagasse_HAD, *q* = 0.0116; bagasse_LYO, *q* = 0.0201), and *Erysipelotrichaceae* UCG-003 (bagasse_HAD, *q* = 0.0002; bagasse_LYO, *q* = 0.2636) genera than that of the fresh bagasse. Moreover, heat treatment of the bagasse gave rise to an increase in the abundance of several SCFA-producer genera such as *Eubacterium* (*p* = 0.0495, *q* = 0.1424), *Coprococcus* (*p* = 0.0014, *q* = 0.0118), *Ruminococcus* (*p* = 0.0194, *q* = 0.0835), *Agathobacter* (*p* = 0.0004, *q* = 0.005) or *Subdoligranulum* (*p* = 0.0135, *q* = 0.0619) after the colonic fermentation.

## 4. Discussion

### 4.1. Effect of Processing on Antiradical Capacity and Phenol Content

The polyphenol composition of almond (*Prunus dulcis*) is significantly affected by the harvest time, environmental factors, agriculture practices, ripening, and variety [25]. As presented above, catechin was also the most abundant polyphenol found in almond kernels by previous studies [26,27,28]. However, data found in the literature referring to polyphenol contents in almond kernel were variable, probably due not only to agronomic conditions, maturity stage, and variety, but also to the extraction solvent and methodology [25]. In almond bagasse, water extraction from the kernel could solubilize polyphenolic components to varying degrees depending on their hydrophilicity. For a more accurate investigation, the compounds present in fresh and/or processed bagasse should be identified by NMR spectroscopy and be compared to those found in the integral product. Furthermore, EPR methodology could be used to determine both oxidative stress and antioxidant capacity. These are the main limitations of this study.

The bagasse had a high humidity and water activity, and needed to be stabilized to prevent degradation and prolong shelf-life so that it could be recovered and reused. Hot air drying at two temperatures and lyophilization were carried out. In both dehydration treatments, the reduction in polyphenolic compounds could be due to the chemical and enzymatic reactions induced in the product by the temperature, or be facilitated by structural changes and interactions that occurred in the samples. The main structural changes induced by dehydration treatments were cellular turgor loss, alteration of the middle lamella, altered cell wall strength, changes in the volume fractions of gas and liquid, as well as changes in the size and shape of the samples [29]. They all could facilitate the interaction between enzymes and the wide variety of bioactive components, such as polyphenols and other macromolecules acting as affective activators of oxidation reactions and located in the different cell structures. For example, George et al. [30] showed that low concentrations of the free fatty acids such as oleic acid, linoleic acid, and arachidonic acid were effective activators of epicatechin oxidation in vitro, suggesting that these endogenous free fatty acids may play a role in the polyphenol oxidase-mediated browning of avocado fruit in vivo. In lyophilization, the freezing and sublimation of water degraded cellular structure and promoted the formation of a porous structure. However, the absence of high temperatures and oxygen could reduce degradation. In hot air-drying treatments, structural damage and oxygen presence at a high temperature resulted in a higher degradation. Nevertheless, the difference between 60 and 70 °C could be decisive in the inactivation of enzymes involved in some of the degradation reactions. Regarding the interaction with other molecules, dehydration has been shown to increase polyphenolic compounds, despite some degradation, because it may improve extraction and lead to a greater release of these compounds [10]. In almond bagasse, the macronutrient composition, consisting mainly of fiber and fat, could interact with the polyphenols and prevent their release after processing.

The differences found between the antiradical capacity measurements were probably due to the differences between the bioactive compounds and the radical affinity of ABTS or DPPH. In fact, the DPPH method was found to be more sensitive to hydrophobic antiradicals, while the ABTS method was found to be more sensitive to hydrophilic antiradicals [31].

### 4.2. In Vitro Gastrointestinal Digestion and Colonic Fermentation

As described above, there is generally a progressive increase in some polyphenol contents and antiradical capacities as gastrointestinal digestion progresses. A possible hypothesis that could explain the results obtained would be based on the interactions established among bioactive compounds and macromolecules. Bioactive compounds can be free or bonded. Structural changes and oxidation reactions induced by dehydration treatment would have a stronger effect in free compounds. Thus, the mixture of enzymes and pH variations at gastric stages affected only the free bioactive compounds of fresh samples but not those remaining bound in the treated ones. However, the mixture of enzymes and pH variations at the intestinal stages could affect the interactions of the bioactive compounds, promoting the release of these treated or fresh compounds. It seems that there was a progressive structural degradation that started to release the bioactive compounds after initial degradation. At the colon stage, the gut microbes metabolized polyphenols leading to smaller size metabolites that were more bioavailable than the original ones [32]. The resulting metabolites showed high bioactivity [33]. This effect can be explained considering the reaction sequences suffered by the polyphenols in the gut, promoted by the enzymes produced by microbiota. The sequence followed to convert polyphenols into smaller metabolites is well described in the literature [32]. The presence of an ester moiety in the hydroxycinnamic acid reduced their intestinal absorption and reached the colon where the gut microbial esterase performed deconjugation and released the free acid from esters. Hydroxycinnamic acid metabolism by the gut microbiota involved many other biochemical transformations such as (de)hydrogenations, (de)methylations, (de)hydroxylations, b-oxidations, etc., leading to diverse metabolites detected in human plasma and urine in individuals with a diet rich in hydroxycinnamic acids [34]. Glycosidic deconjugation to release aglycones, the hydrogenation of the double bond, opening of the C-ring, and catechol-dehydroxylations are the main metabolic pathways of flavonoids in the colon to release smaller molecules with the capability to be absorbed [32].

### 4.3. Fermentative Microbiota Analysis

In a previous work [10], it was indicated that the substrates affected the microbial community growing during the fermentation. Thus, the bacterial communities that grew on both fresh and treated substrates presented a significantly different structure to the residual bacterial population of the fermentation controls. Additionally, the dehydration of the bagasse has a clear effect on the composition of the in vitro fermentative microbiota. Interestingly, the air-dried technique benefits the growth of several SCFA-producer genera. The great abundance of the Butyrivibrio genus detected after the fermentation of the three bagasses, fresh, heat-treated, and freeze-dried, suggested that the composition of the almond substrate favors its growth. Different species of Butyrivibrio are involved in plant polysaccharide breakdown and butyrate production, a short-chain fatty acid that has beneficial health effects for the host [35]. Moreover, the genera Eubacterium, Fusicatenibacter, Romboutsia, Phascolarctobacterium, and Ruminococcus, which presented high abundance after bagasse fermentations, have been also described as fiber-degraders and butyrate-producers [36,37]. Based on comparative genome analysis, Gerritsen et al. [38] showed that the genus Romboutsia encode a versatile array of carbohydrate metabolic capabilities, respectively, carbohydrate utilization producing formate, acetate, lactate, and butyrate. Phascolarctobacterium has been described as an acetate and propionate producer and is positively associated with positive mood in humans [39]. Moreover, Phascolarctobacterium faecium has been related to the tolerance of metformin in type 2 diabetes patients [40]. Likewise, Holdemanella, Allisonella, and Dialister presented with higher abundance after dehydrated-bagasse fermentations. Valles-Colomer et al. [41] described Dialister and Coprococcus as neuroactive genera that are depleted in depression. A study in mice showed that Holdemanella biformis ameliorates hyperglycemia, improves oral glucose tolerance, and restores gluconeogenesis and insulin signaling in the livers of obese mice [42]. Thus, the colonic fermentation of dehydrated bagasses, specially air-dried bagasse, promotes the growth of commensal bacteria described as beneficial to human health.

## 5. Conclusions

Almond bagasse is an interesting by-product, not only for its fiber content, but also for its richness in polyphenols. Its stabilization via a controlled dehydration treatment combined with milling is an alternative for producing functional powdered ingredients for the food industry. Air drying at 60 and 70 °C and lyophilization reduced the content of all the specific polyphenols analyzed. The retained content was very variable depending on the polyphenol considered and the treatment applied. There was no defined trend. The maximum retention (83.3%) was for apigenin -7- glucoside after air drying at 70 °C and the minimum (29.5%) was for sinapic acid after air drying at 60 °C.

In terms of antiradical scavenging activities and total phenolics, each of the treatments considered significantly reduced the levels. However, in vitro digestion and the intestinal and colonic phases, although still reducing the specific polyphenol content in all the powdered products, made it possible to recover much of the antiradical capacity from the fresh product.

The fermentation of almond bagasse, both fresh and dehydrated, promoted the growth of *Butyrivibrio*, which is a genus described as a fiber degrader and butyrate producer. Thus, the increase in the *Butyrivibrio* genus could have implications for human health. Moreover, the dehydration techniques influenced the composition of the in vitro resulting microbial community, with the air-dried method giving rise to a greater abundance of SCFA-producer genera.

It would be necessary to follow up on the polyphenol biotransformation into smaller molecules/secondary metabolites to identify and describe in detail the increase in the antiradical activity and the total phenols, and how this relates to gut microbiota. Moreover, understanding the role of other macronutrients in the bioaccessibility of polyphenols, in their degradation or biotransformation, is of vital importance to provide relevant and holistic information about the product as well as a detailed effect of dehydration treatments and the digestion process.

## Data Availability

The sequence datasets generated during the current study are available in the ENA database with the accession number PRJEB61665.

## Supplementary Materials

The following supporting information can be downloaded at https://www.mdpi.com/article/10.3390/antiox12061229/s1. Figure S1: Bar plot of fold-changes in relative abundance of bacterial genera between feces fermentation and freeze-dried bagasse (bagasse_LYO), air-dried bagasse (bagasse_HAD) and fresh bagasse (bagasse) fermentations. Fold-changes are represented as log2(FC) and negative values indicate enriched genera in bagasse_LYO, bagasse_HAD and bagasse fermentations; Figure S2: Bar plot of fold-changes in relative abundance of bacterial genera between fresh bagasse (bagasse) fermentation and freeze-dried bagasse (bagasse_LYO) and air-dried bagasse fermentations. Fold-changes are represented as log2(FC) and negative values indicate enriched genera in fresh bagasse fermentation; Table S1: Pairwise comparisons between feces and bagasse_LYO, bagasse_HAD or fresh bagasse; Table S2: Pairwise comparisons between fresh bagasse and bagasse_HAD or bagasse_LYO.
