# Supplementary material for: Almond (Prunus dulcis) Bagasse as a Source of Bioactive Compounds with Antioxidant Properties: An In Vitro Assessment
Source: Antioxidants (Basel). 2023 Jun 7;12(6):1229. doi: 10.3390/antiox12061229 (PMC10295057; doi:10.3390/antiox12061229)
Supplement: Supplementary file 1 [file antioxidants-12-01229-s001.zip › Supplementary_Figures.zip!/Supplementary_Figures.pdf]

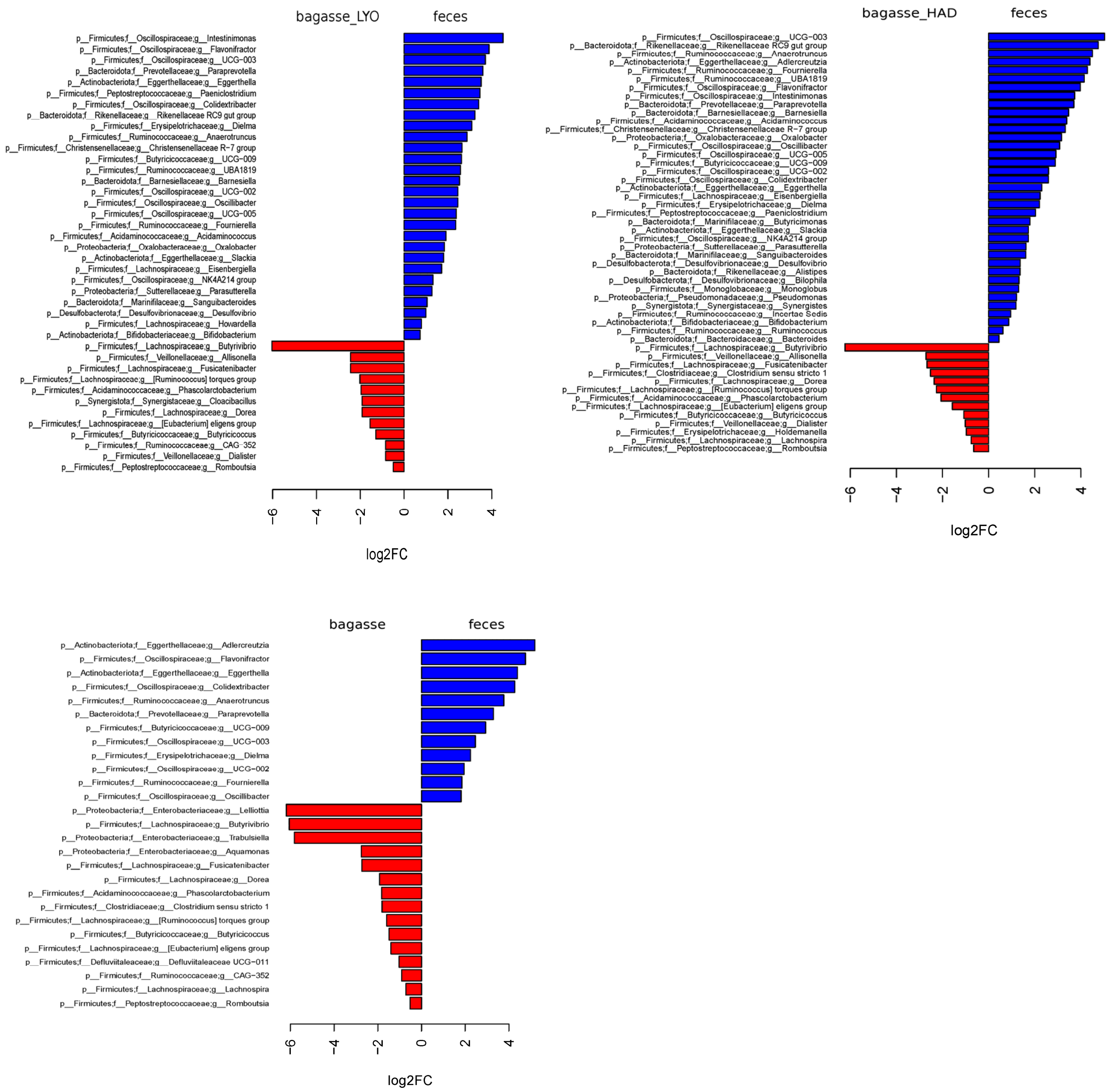

Figure S1. Bar plot of fold-changes in relative abundance of bacterial genera between feces fermentation and freeze-dried bagasse (bagasse_LYO), air-dried bagasse (bagasse_HAD) and fresh bagasse (bagasse) fermentations. Fold-changes are represented as log2(FC) and negative values indicate enriched genera in bagasse_LYO, bagasse_HAD and bagasse fermentations.

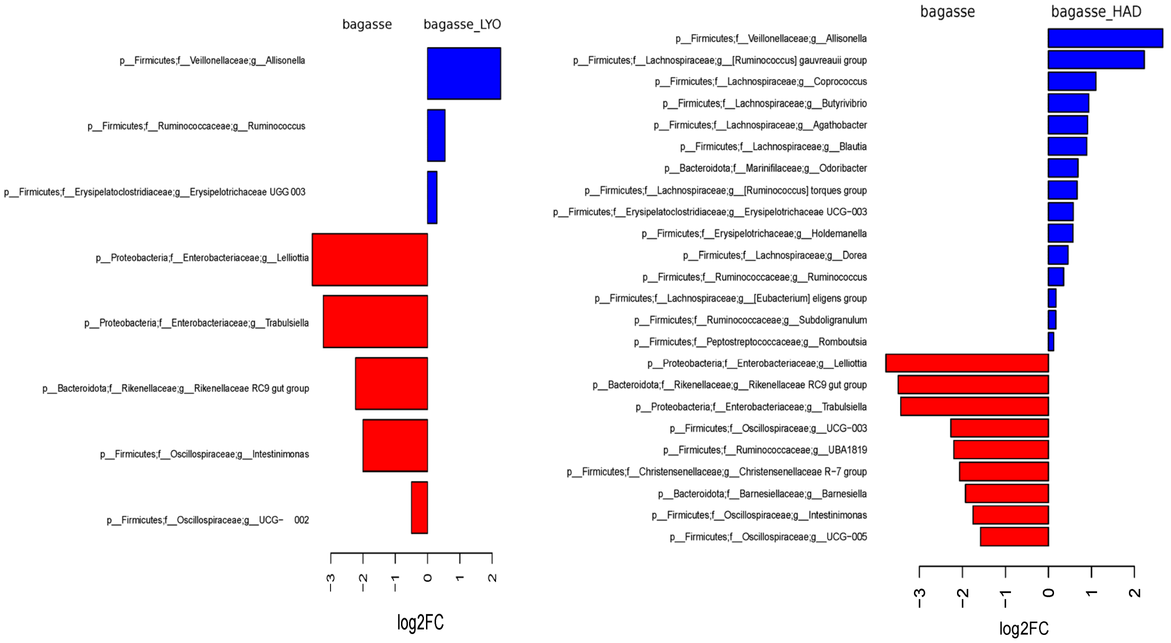

Figure S2. Bar plot of fold-changes in relative abundance of bacterial genera between fresh bagasse (bagasse) fermentation and freeze-dried bagasse (bagasse_LYO) and air-dried bagasse fermentations. Fold-changes are represented as log2(FC) and negative values indicate enriched genera in fresh bagasse fermentation;.
