# Supplementary material for: Almond (Prunus dulcis) Bagasse as a Source of Bioactive Compounds with Antioxidant Properties: An In Vitro Assessment
Source: Antioxidants (Basel). 2023 Jun 7;12(6):1229. doi: 10.3390/antiox12061229 (PMC10295057; doi:10.3390/antiox12061229)
Supplement: Supplementary file 1 [file antioxidants-12-01229-s001.zip › Supplementary_Tables.zip!/Supplementary_Tables.pdf]

Supplementary Tables:

Table S1: Pairwise comparisons between feces and bagasse_LYO, bagasse_HAD or fresh bagasse

	Bagasse_LYO		Bagasse_HAD		Fresh Bagasse	
**Genus**	p-value	q-value	p-value	q-value	p-value	q-value
Eubacterium eligens group	1.0932E-06	5.9345E-06	7.3608E-10	5.0804E-09	0.0023	0.0107
Ruminococcus torques group	8.2754E-07	4.8379E-06	2.5295E-08	1.4093E-07	0.0021	0.0107
Butyrivibrio	8.8966E-38	3.3807E-36	2.0238E-57	3.1572E-56	1.2626E-14	3.3249E-13
Dorea	0.0001	0.0005	4.3984E-08	2.2872E-07	0.0028	0.0117
Fusicatenibacter	0.0057	0.0145	0.0001	0.0003	0.0195	0.0617
Butyricicoccus	0.0082	0.0195	0.0062	0.0110	0.0086	0.0325
Ruminococcaceae CAG-352	0.0014	0.0046	ns	ns	0.0062	0.0245
Romboutsia	0.0363	0.0708	0.0007	0.0016	0.0377	0.1103
Phascolarctobacterium	6.5179E-14	5.5040E-13	1.9645E-07	9.5767E-07	7.8464E-05	0.0006
Allisonella	0.0033	0.0093	0.0053	0.0096	ns	ns
Dialister	0.0447	0.0850	0.0101	0.0172	ns	ns
Cloacibacillus	0.0173	0.0364	ns	ns	ns	ns
Holdemanella	ns	ns	0.0028	0.0055	ns	ns
Clostridium sensu stricto 1	ns	ns	4.4886E-05	1.2504E-04	0.0125	0.0429
Lachnospira	ns	ns	0.0176	0.0286	0.0342	0.1039
Defluviitaleaceae UCG-011	ns	ns	ns	ns	0.0114	0.0409
Aquamonas	ns	ns	ns	ns	3.5344E-07	3.9169E-06
Lelliottia	ns	ns	ns	ns	1.2359E-42	9.7634E-41
Trabulsiella	ns	ns	ns	ns	3.5344E-07	3.9169E-06

ns; not significative

Table S2: Pairwise comparisons between fresh bagasse and bagasse_HAD or bagasse_LYO

	**Bagasse_LYO**		**Bagasse_HAD**	
**Genus**	p-value	q-value	p-value	q-value
Romboutsia	ns	ns	0.0302	0.1041
Subdoligranulum	ns	ns	0.0135	0.0619
Eubacterium eligens group	ns	ns	0.0495	0.1424
Ruminococcus	0.0015	0.0201	0.0012	0.0116
Dorea	ns	ns	0.0047	0.0335
Holdemanella	ns	ns	0.0073	0.0419
Erysipelotrichaceae UCG-003	0.0280	0.2636	6.7429E-06	0.0002
Ruminococcus torques group	ns	ns	0.0194	0.0835
Odoribacter	ns	ns	0.0355	0.1112
Blautia	ns	ns	0.0064	0.0401
Agathobacter	ns	ns	0.0004	0.0050
Butyrivibrio	ns	ns	0.0339	0.1112
Coprococcus	ns	ns	0.0014	0.0118
Ruminococcus gauvreauii group	ns	ns	0.0084	0.0446
Allisonella	2.5180E-16	4.1547E-15	1.1345E-05	0.0002

ns; not significative
